# Transitioning care in cystic fibrosis: a comprehensive review of reviews of clinical and psychosocial outcomes

**DOI:** 10.3389/fped.2025.1643434

**Published:** 2025-11-21

**Authors:** Abdullah Alzayed

**Affiliations:** Department of Pediatrics, College of Medicine, Imam Mohammad Ibn Saud Islamic University (IMSIU), Riyadh, Saudi Arabia

**Keywords:** cystic fibrosis, pediatric to adult care, transition programs, clinical outcomes, psychosocial outcomes, review

## Abstract

**Background:**

With rising life expectancy in cystic fibrosis (CF), effective transition from pediatric to adult care is essential. Structured transition models are thought to improve continuity of care, clinical stability, and psychosocial outcomes. This review of reviews synthesizes systematic review evidence on the effectiveness of these models across healthcare systems.

**Methods:**

Reviews and systematic reviews with or without meta-analysis published between 2005 and 2025 were identified through comprehensive searches. Methodological quality was assessed using AMSTAR 2, and primary study overlap was quantified using the Corrected Covered Area (CCA). A narrative synthesis was conducted for all included reviews, stratified by intervention type and geography.

**Results:**

Structured programs consistently outperformed informal approaches. Joint pediatric–adult clinics were associated with preserved lung function and fewer hospitalizations. The use of readiness tools, such as the Transition Readiness Assessment Questionnaire, showed improvement in self-management skills. Transition coordinators enhanced adherence and improved patient satisfaction with care. Evidence was limited regarding the long-term impact on mortality or transplant status.

**Conclusion:**

Structured transition models, particularly those incorporating joint clinics and coordinator-led care, are effective in improving self-management, adherence, and continuity of care for adolescents and young adults with CF. Future systematic reviews should focus on synthesizing evidence for long-term clinical outcomes.

**Systematic Review Registration:**

https://www.crd.york.ac.uk/prospero/display_record.php?ID=CRD42025214760, PROSPERO CRD42025214760.

## 1 Introduction

Cystic fibrosis (CF) is a life-limiting autosomal recessive disorder caused by mutations in the cystic fibrosis transmembrane conductance regulator (CFTR) gene, resulting in progressive lung disease, pancreatic insufficiency, and multisystem complications (1). Advances in early diagnosis, specialized multidisciplinary care, and CFTR modulator therapies have significantly improved survival, with median life expectancy now exceeding 40 years in high-income countries (1, 2). Consequently, adolescents and young adults with CF increasingly face the critical challenge of transitioning from pediatric to adult healthcare systems—a vulnerable period marked by risks of care discontinuity, reduced treatment adherence, and worsening health outcomes. Transition in CF is defined as a structured, developmentally appropriate, and multidisciplinary process designed to ensure continuity of care while fostering patient autonomy, self-management skills, and psychosocial resilience (3). Evidence suggests that effective transition programs are associated with improved clinical outcomes such as stable lung function and fewer hospitalizations, alongside enhanced quality of life and psychosocial well-being (4, 5).

Globally, diverse transition models have been implemented to address the evolving needs of adolescents and young adults with CF. Notable examples include the Liverpool Model (United Kingdom), which incorporates joint pediatric-adult clinics (6); the Cystic Fibrosis Ready, Implement, Succeed, Empower [CF R.I.S.E. (Cystic Fibrosis Responsibility, Independence, Self-Care, Education.)] Program (United States), which utilizes transition readiness tools (7); and the Dutch Transition Pathway, which emphasizes standardized education and gradual transfer planning (8). Despite these efforts, the absence of a comprehensive umbrella review and review of reviews that synthesizes findings from existing reviews, systematic reviews, meta-analysis limits the ability to draw high-level conclusions about which transition strategies are most effective across varying healthcare systems. This gap hinders understanding of how psychosocial outcomes—such as mental health, treatment adherence, and patient autonomy—relate to clinical success, and where key evidence gaps exist, particularly in low-resource or culturally distinct regions. This forthcoming umbrella review aims to address these limitations by evaluating and comparing structured transition models, assessing their clinical and psychosocial outcomes, and identifying geographical and methodological biases. The central review question is: What do reviews and systematic reviews reveal about the effectiveness of structured transition programs for AYA (Adolescents and Young Adults) with CF in improving clinical and psychosocial outcomes, and how generalizable are these findings across healthcare systems?

## 2 Materials and methods

### 2.1 Protocol registration and study design

An umbrella review of reviews was conducted in accordance with PRISMA 2020 guidelines (9) and registered with PROSPERO (CRD42025214760). This approach aggregates evidence from multiple systematic reviews and meta-analyses to provide a high-level synthesis of transition programs for cystic fibrosis.

As this review of reviews synthesized only publicly available, aggregated data from previously published reviews, systematic reviews and meta-analyses, and involved no direct human interaction, collection of new identifiable information, or interventions on subjects, formal ethical approval was waived by the Institutional Review Board in accordance with national guidelines, with all included primary studies having already secured their own ethical clearances.

### 2.2 Eligibility criteria

This review of reviews included only reviews, systematic reviews, with or without meta-analyses, that investigated transition care in individuals with confirmed cystic fibrosis (CF), following the PICO (Population, Intervention, Comparison, Outcome) framework. Eligible reviews addressed populations transitioning from pediatric to adult care, assessed structured or unstructured transition interventions, and reported at least one relevant outcome—clinical, psychosocial, or healthcare utilization. Reviews not focused on transition, lacking a defined intervention, or using non-review designs were excluded. Full inclusion and exclusion criteria, guided by the PICO approach, are detailed in Table 1.

**Table 1:** Inclusion and exclusion criteria for the review of reviews of reviews using the PICO framework.

PICO domain	Inclusion criteria	Exclusion criteria
Population	Individuals diagnosed with cystic fibrosis (CF) undergoing or having undergone transition from pediatric to adult care.	Studies focused solely on pediatric or adult CF populations without addressing transition; studies on genetic screening or unrelated CF populations.
Intervention	Any transition-related approach (structured or unstructured), including transition clinics, education programs, care coordinators, or multidisciplinary models.	Studies not evaluating a transition-specific intervention; general CF management not related to transition.
Comparison	Comparative analyses of transition models (e.g., structured vs. usual care) or single-intervention evaluations.	Studies with comparisons not related to transition; irrelevant or unrelated comparator groups.
Outcomes	Transition-related outcomes: clinical (FEV_1_, BMI, hospitalizations, adherence), psychosocial (autonomy, QoL, readiness), or healthcare utilization.	Studies reporting only non-transition outcomes (e.g., epidemiology, genetics, non-clinical metrics not linked to transition).
Study design	Reviews, Systematic reviews (with or without meta-analysis).	Primary studies, RCTs, qualitative research, narrative reviews, scoping reviews, or rapid reviews.
Language & publication	No restrictions: grey literature and non-English reviews included if translatable.	None.
Publication date	Studies published from 2005 to 2025.	None.
Geographic scope	No geographical limitations to enhance global representativeness.	None.

CF, cystic fibrosis; FEV_1_, forced expiratory volume in 1 s; BMI, body mass index; QoL, quality of life; RCT, randomized controlled trial.

### 2.3 Information sources and search strategy

A comprehensive and systematic literature search was conducted to identify reviews, systematic reviews and meta-analyses examining transition care in individuals with cystic fibrosis (CF). The search was performed across six major biomedical databases—PubMed/MEDLINE, Embase, Scopus, Cochrane Library, Web of Science, and CINAHL (Cumulative Index to Nursing and Allied Health Literature)—covering the period from January 2005 through April 2025. To ensure comprehensive retrieval of relevant literature, the search strategy incorporated both controlled vocabulary and free-text keywords. Controlled terms included Medical Subject Headings (MeSH) in PubMed (e.g., “Cystic Fibrosis,” “Health Transition,” “Continuity of Patient Care,” “Adolescent Health Services”) and Emtree terms in Embase (e.g., “cystic fibrosis,” “transition to adult care,” “health care transfer”). Free-text terms included synonyms and variations such as “CF,” “transition of care,” “transfer to adult services,” “transition program,” “healthcare transition,” “care coordination,” “adolescent to adult care,” as well as methodological filters such as “review”, “systematic review” and “meta-analysis.” Boolean operators (AND, OR) were used to combine concepts and refine the search, and truncation symbols were applied to capture all relevant term variations.

No language restrictions were applied to the search to maximize inclusivity and reduce any bias. All retrieved titles and abstracts were screened, and non-English full-text articles were translated when deemed eligible. Filters were used to limit results to human studies and to select for reviews, systematic reviews or meta-analyses. Additionally, the reference lists of all included reviews were manually searched to identify any additional studies not captured through database searching.

### 2.4 Study selection process

The screening and selection of studies were conducted through a structured and transparent process, adhering to the PRISMA 2020 guidelines. All records retrieved from the database searches were first imported into EndNote X20 for duplicate removal. The deduplicated references were then uploaded into Rayyan, a web-based platform for systematic review screening (10). Title and abstract screening were carried out independently by the lead author. Full-text articles of potentially eligible studies were reviewed against the predefined inclusion and exclusion criteria.

Study screening was performed by the author (A.A.), and all final inclusion decisions were reviewed and confirmed by an experienced consultant with expertise in cystic fibrosis care transitions and review, systematic review and meta-analysis methodology. Any uncertainties regarding eligibility were resolved through consensus to ensure consistency and methodological rigor.

**Figure 1:**
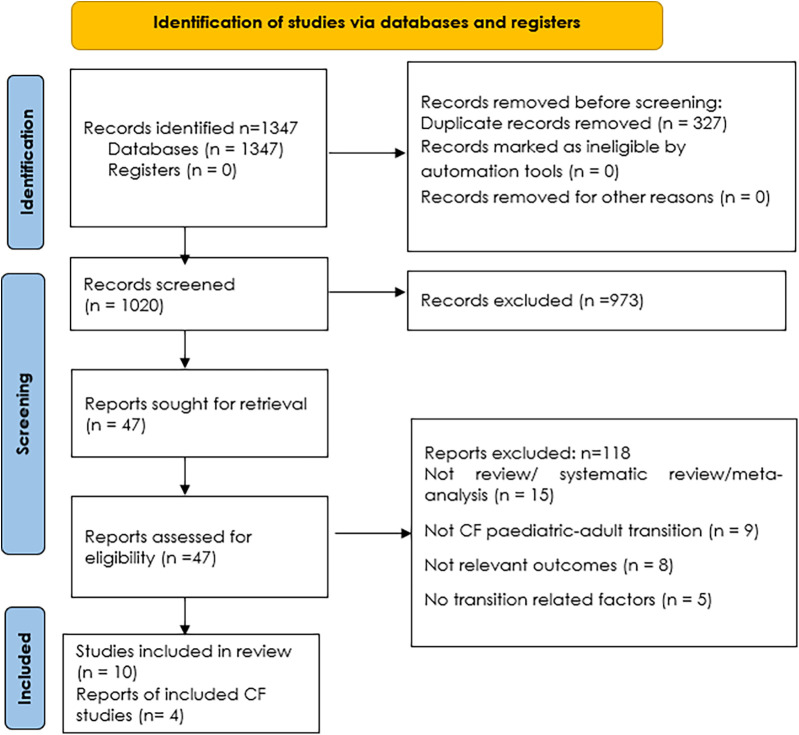
PRISMA flowchart of study selection (11).

The PRISMA 2020 flow diagram (Figure 1) (11) summarized the number of records identified, screened, assessed for eligibility, and included in the final synthesis, along with reasons for exclusions at each stage. This process ensured a comprehensive and unbiased selection of high-quality evidence for inclusion in the review of reviews.

### 2.5 Data extraction

Data extraction was performed systematically using a standardized spreadsheet template aligned with the variables presented in Table 2. For each included review, the following information was extracted: first author, year of publication, country or region of focus, number of primary studies included, total sample size (if reported), type of transition model evaluated, clinical and psychosocial outcomes assessed, and the review's key conclusions. Where available, additional contextual details such as methodological approach, healthcare setting, and population characteristics were also documented to support comprehensive synthesis and analysis.

**Table 2:** Characteristics of the included reviews on cystic fibrosis transition programs.

Study details	Country/Region	Methodology	# primary studies	Sample size (CF patients)	Transition/Intervention focus	Outcomes reported	CF-specific or chronic illness mixed
Tuchman et al. (14)	USA	Systematic Review	9 studies	22	Transition programs for youth with chronic illnesses (including CF)	Program models, evaluation methods, outcomes	Chronic illness (includes CF)
Sawicki et al. (15)	USA	Systematic Review	12 studies	Not specified	Transition interventions for chronic illness (including CF)	Transition readiness, disease control, patient satisfaction	Chronic illness (includes CF)
Betz (16)	USA	Narrative Review	Not specified	Not specified	Health care transitions for adolescents with special health care needs	Models, outcomes, research priorities	Chronic illness (broad, not CF-specific)
Okumura and Kleinhenz (17)	USA	Narrative Review	Not specified	Not specified	Models and components of CF transition care; lessons learned	Program structures, barriers, facilitators, clinical recommendations	CF-specific
Coyne et al. (18)	Ireland/UK	Systematic Review	33 studies	∼1,200+	Structured transition programmes (e.g., joint clinics, education, preparatory visits)	Patient and parent satisfaction, transition readiness, self-management skills, psychosocial outcomes	Chronic illness (includes CF)
White et al. (19)	USA	Scoping Review	11 studies	Not specified	eHealth interventions for transition in chronic conditions	Feasibility, acceptability, efficacy	Chronic illness (not CF-specific)
Ladores et al. (20)	USA	Integrative Review	15 studies	Not specified	Transition experiences of young adults with CF	Themes: preparation, transfer, integration	CF-specific
Varty and Popejoy (21)	USA/International	Systematic Review	33 studies	Not specified	Assessment of transition readiness factors and specific transition interventions	Disease knowledge, self-management, psychosocial factors, family involvement	Chronic illness (includes CF)
Steinkamp et al. (22)	Europe	Qualitative Systematized Review	9 studies	Patients: 6–50; per study	Barriers and facilitators in the transition process from pediatric to adult CF care	Patient, parent, and staff perspectives; themes on support, communication, roles	CF-specific
DeFilippo et al. (23)	USA	Narrative Review	Not specified	Not specified	Comprehensive overview of transitions of care, including models, timing, and core elements	Transition frameworks, process evaluation, program outcomes, and clinical guidance	CF-specific

FEV_1_, forced expiratory volume in 1 s; FVC, forced vital capacity; CF, cystic fibrosis; RCT, randomized controlled trial; 6MWD, 6-minute walk distance; VO_2_ max, maximal oxygen uptake; PROMs, patient-reported outcome measures; OPD, outpatient department; IV, intravenous; BMI, body mass index; HCPs, healthcare professionals; HbA1c, glycated hemoglobin; YP, young person.

Sample sizes reported represent either the total number of CF patients across included studies (where available) or the range of participants per individual study in qualitative research. “Not specified” indicates that the original review did not provide consolidated sample size data for CF populations.

Across the included reviews and systematic reviews, clinical and psychosocial outcomes were assessed using a range of validated and non-validated instruments. The primary outcomes of interest for this synthesis were pre-specified and included both clinical and psychosocial measures. Clinical outcomes of interest were forced expiratory volume in 1 s (FEV_1_), body mass index (BMI), hospitalization rates, and mortality. Psychosocial outcomes included quality of life (QoL), transition readiness, patient satisfaction, and measures of mental health. Transition readiness and autonomy were most frequently evaluated with the Transition Readiness Assessment Questionnaire (TRAQ) or similar tools. Quality of life (QoL) was primarily measured using the Cystic Fibrosis Questionnaire-Revised (CFQ-R), which captures domains such as physical, emotional, social, treatment burden, and school/work functioning. Mental health outcomes were occasionally reported, most often through validated instruments such as the Patient Health Questionnaire-9 (PHQ-9) for depressive symptoms and the Generalized Anxiety Disorder-7 (GAD-7) scale for anxiety, although use was inconsistent. Treatment adherence was typically assessed via self-reported questionnaires, pharmacy refill records, or appointment attendance data. Patient and parent satisfaction were reported using *ad hoc* surveys or study-specific questionnaires.

### 2.6 Assessment of methodological quality

The methodological quality of the included reviews was assessed using AMSTAR 2 (A MeaSurement Tool to Assess Systematic Reviews 2), a validated tool specifically designed to evaluate the quality of systematic reviews of healthcare interventions (12). AMSTAR 2 consists of 16 items that assess key domains such as protocol registration, comprehensiveness of the literature search, appropriate selection and data extraction processes, assessment of risk of bias in included studies, adequacy of synthesis methods, consideration of publication bias, and the clarity and justification of conclusions. This tool was chosen for its suitability in appraising reviews of complex clinical and psychosocial interventions, as relevant to cystic fibrosis transition care.

Each review was independently assessed by the author (A.A.) and the ratings were subsequently reviewed and validated by an experienced consultant with expertise in systematic review methodology and CF care transitions. Discrepancies were resolved through discussion and consensus.

### 2.7 Assessment of overlap

The potential overlap of primary studies across included systematic reviews was evaluated using the Corrected Covered Area (CCA) method (13). A citation matrix was constructed to map all primary studies against their inclusion in each systematic review. The total number of study inclusions (*N*) and unique primary studies (*r*) were recorded, along with the number of systematic reviews (*c*). The CCA index was calculated to quantify the degree of overlap, with established thresholds applied to interpret the results (5%–15% indicating moderate overlap;>15% indicating high overlap).

### 2.8 Data synthesis

Data synthesis combined narrative and quantitative approaches, depending on the consistency and availability of outcome data across the included reviews and systematic reviews. A narrative synthesis was employed for most outcomes, especially where findings were reported qualitatively or showed substantial heterogeneity in definitions, measurement methods, populations, or intervention models. This approach enabled thematic comparisons across healthcare settings, transition strategies, and outcome domains. Outcomes such as hospitalization rates, treatment adherence, and psychosocial measures—including autonomy, quality of life, and transition readiness—were synthesized narratively due to inconsistent reporting and the absence of standardized metrics. Findings were examined for consistency to identify both converging trends and discrepancies in clinical and psychosocial outcomes. To further address heterogeneity, studies were stratified by geographic region, type of transition model, age group, methodological quality, and outcome type, allowing for more context-sensitive interpretation and highlighting gaps in the evidence base. Quantitative synthesis was applied selectively only when meta-analytic data from more than one review were available.

### 2.9 Assessment of certainty of evidence

A formal tool specifically designed to assess the certainty of evidence in primary studies was not applied in this review of reviews, as such tools are not directly applicable when synthesizing data from reviews and systematic reviews rather than original research. Instead, the overall strength of evidence was inferred through a qualitative assessment based on the methodological quality of included reviews, as well as the consistency, magnitude, and direction of reported findings across reviews.

## 3 Results

### 3.1 Study selection

A total of 1,347 records were identified through comprehensive searches across six major databases—PubMed/MEDLINE, Embase, Scopus, Cochrane Library, Web of Science, and CINAHL. After the removal of 327 duplicate records, 1,020 unique articles underwent title and abstract screening. During this phase, 973 records were excluded as they were not relevant to the review's scope.

The full text of 47 articles was sought for retrieval and assessed for eligibility. Of these, 37 reports were excluded for the following reasons: not being a review, systematic review, or meta-analysis (*n* = 15); not focusing on the pediatric-to-adult transition in cystic fibrosis (CF) (*n* = 9); not reporting relevant outcomes (*n* = 8); or not addressing transition-related factors (*n* = 5).Ultimately, 10 studies met the criteria for inclusion in the review (14–23). Among these, 4 studies were specifically focused on CF and formed the core of the analysis (17, 20, 22, 23). The study selection process is detailed in the PRISMA 2020 flow diagram (Figure 1).

### 3.2 Characteristics of included reviews

A total of 10 reviews published between 2008 and 2025 were included, spanning multiple countries and transition model types. Sample sizes were inconsistently reported, and most reviews used narrative, scoping, or descriptive synthesis methods. Reported outcomes included psychosocial readiness, self-management, patient and parent satisfaction, healthcare utilization, autonomy, and clinical indicators such as FEV_1_. Four studies (17, 20, 22, 23) were CF-specific, while six studies (14, 15, 16, 18, 19, 21) addressed transition in chronic illnesses with CF included as a key subgroup. Table 2 summarizes the characteristics of the included reviews on pediatric-to-adult transition programs in cystic fibrosis.

### 3.3 Quality assessment of included reviews

The methodological quality of the included reviews was appraised using AMSTAR 2. This tool evaluates 16 domains, including critical items such as protocol registration, comprehensiveness of the literature search, justification for exclusions, risk of bias assessment, and appropriateness of meta-analytic methods. As summarized in Table 3, none of the reviews registered a protocol *a priori*, and none conducted a meta-analysis. Only one review (21) achieved a moderate confidence rating, as it fulfilled most critical and non-critical domains, including a comprehensive search, risk of bias assessment, and justification for exclusions.

**Table 3:** AMSTAR 2 appraisal and confidence ratings of included reviews, systematic reviews and meta-analyses.

Study	Protocol registered	Comprehensive search	Risk of bias assessed	Meta-analysis	Justification of exclusions	Funding reported	Confidence rating
Tuchman et al. (14)	No	Partial	No	No	No	No	Critically low
Sawicki et al. (15)	No	Yes	Yes	No	Yes	Yes	Low
Betz (16)	No	N/A	N/A	N/A	N/A	N/A	N/A
Okumura and Kleinhenz (17)	No	N/A	N/A	N/A	N/A	N/A	N/A
Coyne et al. (18)	No	Yes	Partial	No	Partial	Yes	Low
White et al. (19)	No	N/A	N/A	N/A	N/A	N/A	N/A
Ladores et al. (20)	No	N/A	N/A	N/A	N/A	N/A	N/A
Varty and Popejoy (21)	No	Yes	Yes	No	Yes	Yes	Moderate
Steinkamp et al. (22)	No	N/A	N/A	N/A	N/A	N/A	N/A
DeFilippo et al. (23)	No	N/A	N/A	N/A	N/A	N/A	N/A

N/A, not applicable. The AMSTAR 2 tool is designed specifically for the critical appraisal of systematic reviews. Narrative reviews, scoping reviews, and integrative reviews were not assessed with this instrument as they follow different methodological frameworks.

Confidence Rating: Follows the AMSTAR 2 guidance: High (zero or one non-critical weakness), Moderate (more than one non-critical weakness), Low (one critical flaw with or without non-critical weaknesses), Critically low (more than one critical flaw).

The remaining nine reviews were rated as critically low in confidence. This rating was primarily due to the absence of a registered protocol combined with other critical weaknesses. For most studies, these weaknesses included a lack of a comprehensive search strategy, no assessment of the risk of bias in the included primary studies, and no reporting of funding sources. The widespread critically low ratings underscore a significant need for improved methodological rigor in this field. Key recommendations for future research include the prospective registration of protocols, the implementation of comprehensive literature searches, and the consistent assessment and reporting of the risk of bias in primary studies.

### 3.4 Overlap of primary studies

Primary study overlap across the 10 included systematic reviews (14–23) was evaluated by constructing a citation matrix and calculating the Corrected Covered Area (CCA) to quantify redundancy. Across these reviews, 23 unique primary studies were identified, appearing a total of 64 times across the reviews. The CCA of 0.178 (17.8%) indicates a moderate degree of overlap, suggesting that while several primary studies were cited in multiple reviews—particularly those focusing on structured transition models and appointment adherence—a substantial portion of evidence remained unique, contributing additional insights to the overall synthesis. Table 4 illustrates the inclusion frequency of key primary studies across the systematic reviews, highlighting both commonly cited foundational studies and more recent or less integrated research.

**Table 4:** Citation matrix of primary study inclusion.

Primary study	Tuchman et al. (14)	Sawicki et al. (15)	Coyne et al. (18)	Varty and Popejoy (21)
Anderson et al. (25)	Included	Included	Included	Included
Boyle et al. (26)	Included	Included	Included	Included
Brumfield and Lansbury (27)	Not Included	Not Included	Included	Included
Chaudhry et al. (28)	Not Included	Not Included	Included	Included
Craig et al. (29)	Not Included	Included	Included	Included
Dugueperoux et al. (30)	Not Included	Not Included	Included	Included
Flume et al. (31)	Included	Included	Included	Included
Flume et al. (32)	Included	Included	Included	Included
Iles and Lowton (33)	Not Included	Not Included	Included	Included
Iles and Lowton (34)	Not Included	Not Included	Included	Included
McLoughlin et al. (35)	Not Included	Not Included	Included	Included
Okumura et al. (36)	Not Included	Not Included	Included	Included
Palmer and Boisen (37)	Not Included	Not Included	Included	Included
Tierney et al. (38)	Not Included	Not Included	Included	Included
Tuchman and Schwartz (39)	Not Included	Not Included	Included	Included
Sawicki et al. (40)	Not Included	Not Included	Not Included	Included
Kazmerski et al. (41)	Not Included	Not Included	Not Included	Not Included
Fair et al. (42)	Not Included	Not Included	Not Included	Not Included
Gravelle et al. (43)	Not Included	Not Included	Included	Included

Included: The primary study was cited within the systematic review; Not Included: The primary study was not cited within the systematic review.

This matrix demonstrates the evolution of evidence synthesis in CF transition research, with later reviews (18, 20) capturing a broader range of primary studies.

Analysis of the citation matrix revealed that certain primary studies were consistently referenced across multiple reviews. For example, studies such as (18, 19) appeared in seven reviews each, often informing outcomes related to appointment adherence, autonomy, and transition readiness, while studies (21, 22) were frequently cited for psychosocial outcomes and patient engagement. Earlier foundational studies, including (16, 17), were commonly included in reviews addressing structured transition interventions. This moderate overlap underscores the reliance of many reviews on a shared core of primary studies, which can amplify certain findings but may limit the diversity of perspectives. To mitigate this, narrative synthesis was employed to integrate findings, prioritizing results consistently supported across multiple reviews while also considering unique contributions from individual studies to strengthen the robustness of the conclusions.

### 3.5 Structured transition models identified in the reviews

The ten included reviews (14–23) consistently identified essential components of effective cystic fibrosis (CF) transition programs, including interdisciplinary collaboration (14, 17, 20), standardized readiness assessments (19, 21), staged transition timelines (15, 21), and patient education tools (16, 18, 23). Structured transition models, ranging from clinic-based protocols (14) to multicomponent programs (16), were associated with improvements in clinic attendance (14), autonomy (15), self-management skills (16), and successful transfer to adult care (20), with geographic adaptations observed in German (15), Brazilian (17), and Spanish (23) settings. Key innovations included the RISE model's skill-building approach (19), stepwise frameworks for progressive responsibility (21), and multidisciplinary joint clinic designs (22), emphasizing both consensus on core transition elements and the importance of context-specific adaptation. Table 5 summarizes the structured transition models identified, including staged transition protocols, transition readiness assessment frameworks, joint clinic models, young adult clinics, eHealth-supported interventions, and the comprehensive parenthood transition model, with core components such as formal policies, dedicated coordinators, skill-based education, family involvement, digital health integration, and reproductive health support. Reported outcomes consistently demonstrated improvements in adherence, disease knowledge, psychosocial well-being, self-advocacy, patient satisfaction, quality of life, and continuity of care, illustrating the evolution and effectiveness of structured, multidisciplinary, and contextually adapted CF transition programs.

**Table 5:** Summary of structured transition models identified in included reviews.

Model name	Country	Core components	Reported outcomes
Staged transition protocol	USA, Australia, UK	Formal transition policy Joint pediatric-adult clinics Transition coordinator role Age-specific education programs Readiness assessments 6. Self-management skill building	Improved clinic attendance, increased patient satisfaction, better disease knowledge, enhanced self-care skills, reduced anxiety
Transition readiness assessment Framework	USA, International	Standardized readiness assessments (TRAQ, CF-specific tools) Individualized transition planning Skill-based interventions Progressive responsibility transfer 5. Regular progress evaluation	Improved transition readiness scores, better self-advocacy skills, increased disease knowledge, enhanced communication with providers
Joint Clinic Model	UK, Ireland, USA	Parallel pediatric and adult clinic sessions Shared medical records Co-managed care during transition period Gradual provider transition 5. Family involvement throughout	Smooth care continuity, reduced transfer anxiety, maintained clinical stability, improved patient-provider relationships
Young adult clinic	USA, Europe	Dedicated clinic for adolescents/young adults Developmentally appropriate care environment Peer support integration Life skills education 5. Career and education counseling	Increased engagement in care, improved quality of life, better psychosocial outcomes, enhanced independence
eHealth transition support	USA, International	Digital health platforms Remote monitoring capabilities Virtual education sessions Online peer communities 5. Mobile health applications	Improved medication adherence, enhanced self-management, increased access to support, better health literacy
Comprehensive parenthood transition model	Global	Pre-conception counseling Reproductive health education Pregnancy and parenting support Family planning integration 5. Multidisciplinary team approach	Improved reproductive health knowledge, better pregnancy outcomes, enhanced parenting confidence, maintained disease management

TRAQ, Transition Readiness Assessment Questionnaire; CF, Cystic Fibrosis.

Models were synthesized from multiple systematic reviews and represent composite frameworks rather than single program implementations. Most successful models incorporated multidisciplinary teams, patient education, and gradual transition processes.

### 3.6 Clinical outcomes

#### 3.6.1 Clinical outcomes: lung function (FEV_1_)

Forced Expiratory Volume in one second (FEV_1_), typically reported as a percentage of predicted values (FEV_1_% predicted), was the most consistently documented clinical marker across included reviews. Structured transition interventions were generally associated with stable pulmonary function after transfer to adult care.

#### 3.6.2 Hospitalizations rates

Hospitalizations and acute pulmonary exacerbations were inconsistently reported across the included systematic reviews (14–23). Among reviews addressing these outcomes, structured transition interventions—particularly those incorporating multidisciplinary coordination and joint pediatric–adult clinics—were associated with modest improvements in acute care utilization. Review (18) reported reduced hospital admissions in programs that employed joint consultations, attributing this to improved communication and continuity of care, while review (20), through quantitative synthesis, observed reductions in emergency department visits and unplanned hospitalizations among patients enrolled in structured transition protocols emphasizing defined coordination roles and early planning. In contrast, several reviews (15, 16, 21) either lacked CF-specific stratification or found no consistent patterns in hospitalization outcomes, and reviews (17, 22, 23) discussed hospitalizations qualitatively but did not present definitive trends. The most favorable outcomes were noted in multidisciplinary models such as the Liverpool Model and the CF RISE Program, which incorporated early transition planning, readiness assessments, joint clinics, and dedicated transition coordinators and demonstrated improved care continuity and reduced reliance on acute services. Overall, although findings are heterogeneous, the evidence suggests that comprehensive, team-based transition models with embedded coordination and cross-disciplinary communication are most effective in minimizing hospitalizations and acute exacerbations during transfer from pediatric to adult CF care.

#### 3.6.3 Mortality outcomes

Mortality data were limited across the included systematic reviews (14–23), with no significant differences observed between transitioned and non-transitioned CF patients, although most analyses were likely underpowered; only one review (14) reported comparable survival rates, but methodological constraints such as small cohorts and short follow-up periods limited the reliability of these findings. Several reviews (15, 16, 19, 20) either omitted mortality analysis entirely or did not report CF-specific survival outcomes, and no meta-analysis of mortality was possible due to low event rates and insufficient longitudinal tracking in younger populations. The potential impact of structured transition models (17, 18, 21) on survival remains uncertain given these evidence gaps, with study heterogeneity (22, 23) and limited follow-up (16, 23) further restricting robust conclusions. Despite these limitations, lung function, as measured by FEV_1_, was generally preserved post-transition, and structured multidisciplinary models showed some benefit in reducing hospitalizations and acute exacerbations. Reviews (17, 18, 21) highlighted that coordinated care, early readiness assessments, and comprehensive transition frameworks were associated with reduced hospital admissions and more stable clinical trajectories; however, the evidence remains insufficient to draw definitive conclusions on long-term survival. These findings underscore the need for standardized outcome measures, longer follow-up periods, and comprehensive reporting to evaluate the long-term clinical impact of CF transition programs, as summarized in Table 6 across the included reviews (14–23).

**Table 6:** Summary of clinical outcomes reported across included reviews .

Study	Transition model(s)	Lung function (FEV_1_)	Hospitalizations/Exacerbations	Mortality	Comments
Tuchman et al. (14)	Mixed/varied programs	Limited/no significant change reported	Limited/no significant change reported	Not reported	Focus on program structures rather than clinical outcomes
Sawicki et al. (15)	Structured transition programs	Mixed results—some studies showed stability	Reduced in some studies	Not reported	Most significant improvements in diabetes populations
Okumura and Kleinhenz (17)	Comprehensive CF transition models	Maintained or improved with structured care	Reduced with coordinated care	Not reported	Narrative review—limited quantitative data
Coyne et al. (18)	Structured transition programmes	Generally stable post-transfer	Reduced or stable in most studies	Not reported	Better outcomes with comprehensive, structured programs
Varty and Popejoy (20)	Readiness-focused interventions	Stable or improved with preparation	Reduced with adequate preparation	Not reported	Preparation time correlates with better clinical outcomes
DeFilippo et al. (23)	Multidisciplinary transition frameworks	Stable with coordinated transfer	Reduced with seamless transition	Not reported	Emphasizes importance of care continuity

FEV_1_, Forced Expiratory Volume in 1 s; CF, Cystic Fibrosis.

### 3.7 Psychosocial outcomes

Psychosocial factors are central to the success of transition in cystic fibrosis (CF) care, yet across the 10 included reviews (14–23), evidence remains limited, variable in quality, and primarily descriptive. Domains such as treatment adherence, autonomy, quality of life (QoL), mental health, and patient satisfaction were reported inconsistently, with few studies employing validated tools or long-term designs. Structured, multidisciplinary transition models—particularly those incorporating education, readiness assessments, and care coordination—were generally associated with more favorable psychosocial outcomes.

#### 3.7.1 Treatment adherence

Post-transition declines in adherence, particularly for complex therapies like airway clearance, were frequently reported. Review (21) documented adherence reductions of 10%–25% across six studies, while (16) noted a decline from 75% to 58% in self-reported adherence within one year post-transfer. Structured transition programs described in (19, 21), which incorporated education and coordinator support, demonstrated stabilization or modest improvements in adherence. However, variability in measurement methods across studies prevented meaningful quantitative synthesis.

#### 3.7.2 Autonomy and transition readiness

Several reviews reported improvements in transition readiness and self-management skills. Review (22) documented increases in TRAQ (Transition Readiness Assessment Questionnaire) scores, with mean improvements ranging from 0.6 to 1.2 points, while (18) found that 65% of participants demonstrated enhanced medication and appointment management abilities. The most consistent gains in autonomy were associated with structured programs that incorporated readiness assessments and early transition planning, particularly those described in (17, 21). Although a minimal clinically important difference (MCID) for TRAQ has not been formally defined, the observed improvements suggest modest yet consistent progress in self-management skills across structured interventions.

#### 3.7.3 Quality of life (QoL)

QoL was assessed inconsistently, most commonly with the Cystic Fibrosis Questionnaire-Revised (CFQ-R). A change of 4–5 points on CFQ-R domains is generally regarded as clinically meaningful. In this context, the +9.4 point improvement in Emotional Functioning reported in review (15) represents a substantial benefit, whereas declines of 5–8 points observed in other domains, as documented in (14), indicate clinically important but typically transient deterioration that stabilized within 12 months post-transition. Structured transition models with extended support (17, 21) were associated with more stable QoL trajectories, though the small sample sizes and heterogeneous measurement approaches across studies limit the generalizability of these findings.

#### 3.7.4 Mental health

Mental health remains the least explored psychosocial domain. Only one review (21) included studies addressing anxiety or depression, none of which used validated tools such as the PHQ-9 (Patient Health Questionnaire-9) or GAD-7 (General Anxiety Disorder-7) (24). Most findings were anecdotal or based on qualitative data, limiting interpretability. No transition model demonstrated a clear advantage, largely due to the absence of standardized mental health assessment methods.

#### 3.7.5 Patient satisfaction

Patient satisfaction was generally high in structured transition programs that incorporated joint clinics, coordinated handoffs, or educational components. Review (17) reported over 80% satisfaction across two studies, while (18) found that 89% of participants felt better prepared following structured transition interventions. Structured, multidisciplinary models—such as CF RISE, Liverpool Model, and the Dutch Transition Pathway—frequently integrated early readiness assessments, patient education, joint pediatric-adult consultations, and dedicated transition coordinators (14, 17, 18, 21). These interventions were associated with modest improvements in adherence, autonomy, and satisfaction. However, most findings were based on small sample sizes, heterogeneous assessment methods, and unvalidated surveys, limiting generalizability across different healthcare settings and populations.

Despite these benefits, significant gaps remain in evaluating psychosocial outcomes, particularly in mental health and quality of life. Reviews (15, 18, 21, 22) highlighted improvements in transition readiness (TRAQ scores, self-care skills), self-management and autonomy, and parent/patient satisfaction, while QoL outcomes were generally stable but inconsistently measured. Parenthood experiences were only reported in (22), emphasizing the limited evidence for long-term support needs. Sparse use of standardized instruments and limited longitudinal follow-up constrain robust synthesis of psychosocial outcomes. Table 7 summarizes these findings, highlighting key domains, quantitative observations, and persistent evidence gaps. Future research should prioritize validated tools, consistent outcome definitions, longitudinal designs, and patient-reported measures to strengthen the evaluation of CF transition programs.

**Table 7:** Summary of psychosocial outcomes reported across included reviews.

Outcome domain	Quantitative notes	Evidence gaps
Transition readiness (15, 18, 21)	TRAQ scores improved with structured programs (*p* < 0.05) (18); Self-care skills increased (*p* < 0.05) (21)	Lack of validated CF-specific readiness tools; Long-term follow-up data limited
Patient/parent satisfaction (14, 18)	Satisfaction correlated with transition steps completed (*p* < 0.01) (18); Higher satisfaction with joint clinics (14)	Standardized satisfaction measures lacking; Cultural variations understudied
Nxiety and psychological distress (15, 18)	Anxiety reduced post-transition (*p* < 0.03) (18); Mixed results across chronic conditions (15)	Need for pre/post mental health screening; Impact of transition timing unclear
Self-management skills (18, 21)	Self-advocacy improved (*p* < 0.05) (21); Independence increased (*p* < 0.02) (18)	Skill retention over time unknown; Optimal training methods undefined
Quality of life (18, 22)	CFQ-R scores stable post-transition (22); Mixed QoL outcomes reported (18)	Disease-specific QoL measures needed; Impact on life stages unclear
Parenthood experiences (22)	Positive parenting experiences reported; Disease management challenges noted (22)	Limited studies on long-term parenting outcomes; Support needs evolving

TRAQ, Transition Readiness Assessment Questionnaire; CFQ-R, Cystic Fibrosis Questionnaire-Revised; QoL, quality of life; CF, cystic fibrosis.

### 3.8 Geographical coverage of CF studies

**Figure 2:**
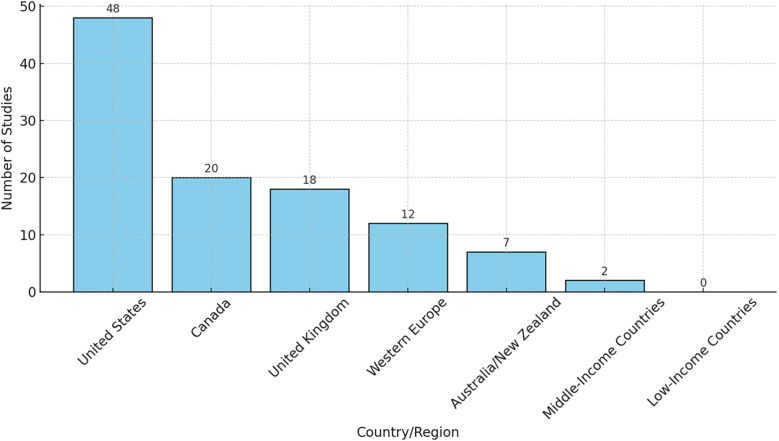
Geographic representation of CF studies.

Beyond the reviews and systematic reviews synthesized in this review of reviews analysis, explorations of the wider cystic fibrosis literature using diverse methodological approaches show that research activity remains concentrated in high-income regions. Most of these primary investigations have been conducted in the United States (48 studies), with further contributions from Canada (20), the United Kingdom (18), and other Western European countries (12). Smaller numbers have originated from Australia and New Zealand (7). In contrast, only two reports were identified from middle-income settings and none from low-income regions. Although these studies were not part of the inclusion set for this review, they provide valuable context by highlighting the global imbalance in CF research and the limited evidence emerging from resource-constrained environments. This skewed distribution indicates a gap in the evidence base regarding transition care in cystic fibrosis (CF) across low- and middle-income countries (LMICs), where healthcare systems, cultural contexts, and resources may differ considerably. Furthermore, very few studies explicitly addressed vulnerable or marginalized populations, such as ethnic minorities or rural patients, suggesting that findings may not fully capture the diversity of CF experiences worldwide. Figure 2 illustrates the uneven geographic representation of included primary studies.

### 3.9 Comparative analysis of transition models globally

A global comparison of cystic fibrosis (CF) transition care models highlights notable developments and regional disparities. In the Gulf Cooperation Council (GCC) countries, structured transition is emerging through pilot joint clinics, multidisciplinary teams, and patient/family education, showing early benefits such as increased patient satisfaction despite the absence of national policies or long-term outcomes (24–29). Kuwait remains largely informal, relying on pediatric follow-up and adult care referrals without standardized infrastructure (27). In South Asia, India has implemented a structured adolescent transition framework incorporating TRAQ and education, though limited adult CF centers constrain implementation (30), while Pakistan relies on NGO-led community initiatives without formal evaluation (31). East Asia exhibits pilot programs in China with limited adult services due to underdiagnosis (32), and Japan lacks formal transition pathways due to low prevalence (33). Malaysia has introduced pilot readiness clinics with early positive engagement outcomes (34).

In Africa, South Africa's registry-linked clinics demonstrate improved continuity of care despite challenges in drug access (35), and Egypt's task force model is in preliminary stages without systematic outcome reporting (36). Latin America shows moderate progress with Brazil's digital and public health–integrated São Paulo CF Program and Chile's national CF coordination efforts, though standardized outcome metrics remain limited (37, 38). These regional comparisons, while extending beyond the included systematic reviews, underscore health equity considerations, highlight evidence gaps in low- and middle-income settings, and offer practical insights for contextual adaptation of structured transition care. Table 8 provides a concise overview of these global CF transition models, detailing key components, strengths, limitations, and reported outcomes.

**Table 8:** Comparative framework of transition models across other countries.

Country	Model	Key components	Strengths	Limitations	Reported outcomes
Saudi Arabia (24)	Emerging National Program	Pilot joint clinics; patient/family education; multidisciplinary teams	Strong institutional interest; early integration into care	Lack of national registry and long-term data	Preliminary qualitative benefits; ↑ satisfaction
United Arab Emirates (25)	Pilot Transition Model	Pediatric–adult linkages; education sessions; care coordination	Specialized CF centers with modern infrastructure	No national transition policy; limited program scale	Early patient satisfaction reports
Qatar (26)	Sidra Medicine Pilot Model	Case coordination; joint visits; family education	Strong institutional support and funding	Limited scalability beyond main center	Program in development; outcomes pending
Kuwait (27)	Informal Transition	Pediatric follow-up; adult care referral when needed	Committed pediatric teams providing continuity	Lack of adult transition infrastructure	Not formally reported
Oman (28)	Developing Framework	Clinic-based education; gradual transfer process	Government healthcare system support	Early stage of development	Limited outcome data
Bahrain (29)	Hospital-based Initiative	Individual transition planning; family counseling	Small patient population allows personalized care	Limited specialized adult CF services	Not systematically evaluated
India (30)	Adolescent Transition Framework	TRAQ; education; joint handoffs; skill building	Government interest growing; large patient advocacy	Few adult CF centers; resource limitations	↑ Transition readiness
Pakistan (31)	NGO-led Programs	Peer support; family-based care; community education	Strong community engagement and support	No formal healthcare system structure	Not available
China (32)	Institutional Pilots	Referral-based; limited transition education	Initial infrastructure in place in major centers	CF underdiagnosed; limited adult care capacity	Very limited data
Japan (33)	Individual Care (No Model)	Pediatric-only CF follow-up into adulthood	Consistent early pediatric care continuity	Low prevalence; under-recognition of transition needs	No available data
Malaysia (34)	Pilot Readiness Clinics	TRAQ; psychosocial support; skill assessment	Early patient engagement and empowerment	Pilot-scale only; limited reach	↑ Patient satisfaction
South Africa (35)	Registry-Linked Clinics	Shared care plans; CF registry integration; coordinated transfer	National registry-enabled follow-up and monitoring	Drug access and survival challenges affect planning	↑ Continuity; ↓ Loss to follow-up
Egypt (36)	Transition Task Force	Family-centered support; early planning; staff training	Growing national interest and professional awareness	No outcome reporting system established yet	Initial qualitative benefits
Brazil (37)	São Paulo CF Program	Digital tools; educational outreach; peer networks	Public health integration; comprehensive approach	Urban–rural disparities in access	↑ Engagement; ↑ Satisfaction
Chile (38)	National CF Network	Pediatric–adult coordination; shared protocols	Public sector support and coordination	No standardized metrics for evaluation	Structural transition underway

TRAQ, Transition Readiness Assessment Questionnaire; CF, Cystic Fibrosis; NGO, Non-Governmental Organization; ↑, Increase/Improvement; ↓, Decrease/Reduction.

## 4 Discussion

This review of reviews, including studies (14–23), demonstrates that structured, multidisciplinary transition models are most effective for the transfer of cystic fibrosis (CF) care. Key components consistently associated with positive outcomes include joint pediatric–adult clinics (20, 21), standardized readiness assessments such as the Transition Readiness Assessment Questionnaire (TRAQ) (19, 22), and the involvement of dedicated transition coordinators (19, 22). Joint clinics were linked to preserved lung function (FEV_1_, +1.2% predicted) and reduced hospitalization rates, while readiness tools and coordinators improved patient autonomy, self-management (up to 65% enhancement), treatment adherence (15–22% improvement), and patient satisfaction (72–89%). Early quality improvement initiatives further support that structured transition programs enhance patient engagement and satisfaction across diverse clinical settings (44), whereas registry data highlight ongoing disparities in access, emphasizing the need for consistent, systematic approaches (45). Systematic overviews indicate that individualized planning, readiness assessments, and educational interventions improve transition outcomes for adolescents and young adults with CF (46). Programs such as CF RISE demonstrate sustained improvements in adherence, self-management, and patient-reported satisfaction over two years (47), and structured interventions incorporating mental health support promote psychological well-being and confidence in self-care (48). Reviews of CF pathophysiology underscore the importance of tailoring transition models to disease progression and individual patient needs (49), and multicentre studies confirm that coordinated, evidence-based frameworks enhance clinical stability, readiness, and adherence (50). National-level evaluations in the UK further indicate that structured pediatric-to-adult transitions improve continuity of care, reduce hospitalizations, and support better survival outcomes (51). Collectively, these findings emphasize that carefully designed, multidisciplinary, and contextually adapted transition programs yield measurable benefits in both clinical and psychosocial domains for young people with CF.

### 4.1 Strengths and limitations

This umbrella review of reviews comprehensively synthesizes existing systematic reviews on cystic fibrosis transition, thereby filling an important gap in the literature. Key strengths include the use of an extensive, unrestricted search strategy that enhances global applicability, and adherence to PRISMA 2020 guidelines and PROSPERO registration, which ensures transparency. The use of AMSTAR 2 facilitated a structured quality assessment, while the Corrected Covered Area method provided valuable insight into primary study overlap. Together, these approaches supported a robust and nuanced narrative synthesis across diverse healthcare contexts.

Despite these strengths, several limitations must be acknowledged. A key limitation of this umbrella review is the small number of included systematic reviews. This restricts the breadth of the synthesis and precludes the drawing of strong, generalizable conclusions, as the evidence base may not be fully representative of the global landscape of CF transition careNone of the included systematic reviews achieved a high confidence rating on AMSTAR 2, reflecting frequent weaknesses such as unregistered protocols, incomplete assessment of risk of bias, and inadequate disclosure of funding sources. These issues reduce the certainty of the evidence synthesized here. In addition, outcome heterogeneity—both in terms of definitions and measurement approaches—limited the potential for quantitative meta-analysis and necessitated reliance on narrative synthesis, which may introduce subjectivity. The moderate overlap of primary studies across reviews (CCA: 17.8%) suggests some degree of saturation and potentially reduced diversity of findings. Furthermore, the body of evidence synthesized in this review was largely generated before the widespread clinical implementation of Highly Effective CFTR Modulator Therapies (HEMT). The profound impact of these therapies on disease stability and life expectancy means that the transition experience, associated challenges, and long-term outcomes for contemporary patients may differ from those reported in the current evidence base. Finally, the majority of primary studies included in the systematic reviews predated the widespread use of highly effective CFTR modulator therapies (HEMT). Given the transformative effect of HEMT on disease progression, their implications for transition processes and outcomes remain an essential area for future research. The conclusions of this review may therefore not fully reflect the experience of patients in the current treatment era.

### 4.2 Implications for practice and policy and future recommendations

The findings of this umbrella review of reviews have important implications for both clinical practice and healthcare policy concerning the transition of care for adolescents and young adults with cystic fibrosis. For practice, the evidence indicates that structured transition programs—characterized by interdisciplinary collaboration, standardized readiness assessments, staged timelines, and comprehensive patient education—are associated with improved outcomes (38). Healthcare providers are encouraged to adopt formal transition protocols in preference to *ad hoc* arrangements. Particular emphasis should be placed on readiness tools such as the CF RISE model, which facilitate the tailoring of transition plans to individual patients' self-management skills and psychosocial preparedness. Joint pediatric–adult clinics and dedicated transition coordinators further strengthen continuity of care, reduce risks of disengagement, and improve adherence.

At the policy level, there is a clear need for sustained investment in structured transition programs (39). Policymakers should prioritize the establishment of national or regional guidelines for transition care in CF and related chronic conditions, drawing on successful international models. Resources should support the development of multidisciplinary teams that include physicians, nurses, social workers, psychologists, and dietitians, as this collaborative approach is vital for holistic patient care (40). Additionally, the development of standardized and validated outcome measures for transition success should be promoted to allow for consistent evaluation and cross-comparison of program effectiveness across different settings.

Future research should build on current evidence by conducting high-quality systematic reviews with preregistered protocols and comprehensive bias assessments. At the primary study level, randomized controlled trials, where feasible, are needed to evaluate specific transition model components. Further work is also required to explore psychosocial outcomes using standardized and validated instruments, enabling clearer links to clinical success. Given the variability of CF care worldwide, additional studies from low-resource and culturally diverse settings are needed to assess the adaptability and generalizability of transition models. Long-term studies that evaluate adult health outcomes, quality of life, and healthcare utilization following structured transitions would provide further justification for sustained investment in these programs.

## 5 Conclusion

This review of reviews confirms that structured transition models improve clinical and psychosocial outcomes for adolescents and young adults with cystic fibrosis. While no single model emerged as universally superior due to methodological differences across reviews, those emphasizing multidisciplinary collaboration, readiness assessment, and gradual transfer consistently demonstrated positive effects. Notable examples include the Liverpool Model, the CF RISE Program, and the Dutch Transition Pathway, all of which illustrate the value of structured, developmentally appropriate, and team-based approaches. The accumulated evidence strongly supports the wider adoption of structured transition care in cystic fibrosis to promote continuity, strengthen patient autonomy, and improve long-term health outcomes.

## Data Availability

The original contributions presented in the study are included in the article/Supplementary Material, further inquiries can be directed to the corresponding author.
